# Supplementary material for: Empirical methods for controlling false positives and estimating confidence in ChIP-Seq peaks
Source: BMC Bioinformatics. 2008 Dec 5;9:523. doi: 10.1186/1471-2105-9-523 (PMC2628906; doi:10.1186/1471-2105-9-523)
Supplement: Additional File 1 — A variety of html documents from the USeq web site detailing the available applications, their best usage, output file type descriptions, command line menus, etc. [file 1471-2105-9-523-S1.zip › USeqUserGuides/JavaDoc.zip!/JavaDoc/trans/main/IntervalReportPrinter.html]

IntervalReportPrinter


|  |  |  |
| --- | --- | --- |
| **Overview** \| **Package** \| **Class** \| **Use** \| **Tree** \| **Deprecated** \| **Index** \| **Help** | |  |
| **PREV CLASS**   **NEXT CLASS** | **FRAMES**    **NO FRAMES**     **All Classes** |  |
| SUMMARY: NESTED \| FIELD \| CONSTR \| METHOD | DETAIL: FIELD \| CONSTR \| METHOD |  |


---


## trans.main Class IntervalReportPrinter

```
java.lang.Object
  trans.main.IntervalReportPrinter
```

---

``` public class IntervalReportPrinter extends java.lang.Object ```

Prints all things contained within an interval to a spreadsheet or a report card.

---

**Constructor Summary**

`IntervalReportPrinter(java.lang.String[] args)`


| **Method Summary** | |
| --- | --- |
| `static double` | `aveMedianAbsDiffSubWinControl(Interval i)`             Calculate maximum median absolute diff of SubWindow control values |
| `static double` | `calculateMeanNumberOligoMatches(Oligo[] oligos)`             Calculates the average number of oligo matches to the genome. |
| `java.lang.String` | `fetchIntervalLine(Interval i, int intervalNumber)` |
| `java.lang.String` | `fetchIntervalReport(Interval i, int intervalNumber)` |
| `void` | `generateIntervalStats(Interval interval)`             Calculate stats on oligos from best window. |
| `static void` | `main(java.lang.String[] args)` |
| `static double` | `maxMedianAbsDiffSubWinTreatment(Interval i)`             Calculate maximum median absolute diff of SubWindow treatment values |
| `static double` | `medianRatioSubWindow(Interval i)`             Calculate Median ratio of SubWindow where the treatments are averaged, and the controls are averaged |
| `static void` | `printDocs()` |
| `void` | `printRatios(Interval interval)`             Used when making mock Intervals from which you want to calculate various oligo based measurements. |
| `void` | `printReport()` |
| `void` | `processArgs(java.lang.String[] args)`             This method will process each argument and assign any new varibles |

**Methods inherited from class java.lang.Object**

`equals, getClass, hashCode, notify, notifyAll, toString, wait, wait, wait`

**Constructor Detail**

### IntervalReportPrinter

```
public IntervalReportPrinter(java.lang.String[] args)
```


**Method Detail**

### printReport

```
public void printReport()
```

---


### generateIntervalStats

```
public void generateIntervalStats(Interval interval)
```

:   Calculate stats on oligos from best window.

---


### calculateMeanNumberOligoMatches

```
public static double calculateMeanNumberOligoMatches(Oligo[] oligos)
```

:   Calculates the average number of oligo matches to the genome.

---


### printRatios

```
public void printRatios(Interval interval)
```

:   Used when making mock Intervals from which you want to calculate various oligo based measurements.

---


### fetchIntervalLine

```
public java.lang.String fetchIntervalLine(Interval i,
                                          int intervalNumber)
```

---


### fetchIntervalReport

```
public java.lang.String fetchIntervalReport(Interval i,
                                            int intervalNumber)
```

---


### printDocs

```
public static void printDocs()
```

---


### processArgs

```
public void processArgs(java.lang.String[] args)
```

:   This method will process each argument and assign any new varibles

---


### main

```
public static void main(java.lang.String[] args)
```

---


### medianRatioSubWindow

```
public static double medianRatioSubWindow(Interval i)
```

:   Calculate Median ratio of SubWindow where the treatments are averaged, and the controls are averaged

---


### maxMedianAbsDiffSubWinTreatment

```
public static double maxMedianAbsDiffSubWinTreatment(Interval i)
```

:   Calculate maximum median absolute diff of SubWindow treatment values

---


### aveMedianAbsDiffSubWinControl

```
public static double aveMedianAbsDiffSubWinControl(Interval i)
```

:   Calculate maximum median absolute diff of SubWindow control values


---


|  |  |  |
| --- | --- | --- |
| **Overview** \| **Package** \| **Class** \| **Use** \| **Tree** \| **Deprecated** \| **Index** \| **Help** | |  |
| **PREV CLASS**   **NEXT CLASS** | **FRAMES**    **NO FRAMES**     **All Classes** |  |
| SUMMARY: NESTED \| FIELD \| CONSTR \| METHOD | DETAIL: FIELD \| CONSTR \| METHOD |  |


---
